# The Skin Photophores of *Chauliodus sloani* Bloch & Schneider, 1801 (Pisces: Stomiidae): A Morphological, Ultrastructural and Immunohistochemical Study

**DOI:** 10.3390/ani15121738

**Published:** 2025-06-12

**Authors:** Mauro Cavallaro, Lidia Pansera, Kamel Mhalhel, Francesco Abbate, Maria Levanti, Maria Cristina Guerrera, Giuseppe Montalbano, Marilena Briglia, Marialuisa Aragona, Rosaria Laurà

**Affiliations:** 1 Zebrafish Neuromorphology Lab, Department of Veterinary Sciences, University of Messina, 98168 Messina, Italy; lidia.pansera@unime.it (L.P.); kamel.mhalhel@unime.it (K.M.); abbatef@unime.it (F.A.); mblevanti@unime.it (M.L.); mguerrera@unime.it (M.C.G.); gmontalbano@unime.it (G.M.); mlaragona@unime.it (M.A.); laurar@unime.it (R.L.); 2 Department of Chemical, Biological, Pharmaceutical and Environmental Sciences, University of Messina, Viale Ferdinando Stagno D’Alcontres 31, 98166 Messina, Italy; 3 Department of Medicine and Surgery, University of Enna Kore, 94100 Enna, Italy

**Keywords:** skin photophores, mesopelagic fishes, structure, ultrastructure, histochemistry, Stomiidae

## Abstract

This work provides a contribution to the understanding of the structure of the photophores in the mesopelagic fish *Chauliodus sloani* (family Stomiidae), which occasionally are stranded along the coasts of the Strait of Messina (Central Mediterranean Sea). The analysis was carried out through the study of the structure and ultrastructure of the ventral photophores, and it also includes an immunohistochemical investigation that offers valuable insights into the function of these organs. Studies on photophores help clarify many aspects of the ecology of this species, which represents an important functional link in the marine food web.

## Simple Summary

The study of bioluminescence and photophores in fish is essential for understanding the evolutionary and ecological strategies of marine organisms, especially in deep-sea environments. Photophores, specialized light-emitting organs, are present in many species of mesopelagic, bathypelagic, and abyssal fish. These organs can serve various functions. Defense against predators: some fish use bioluminescence to confuse or scare off predators by creating visual distractions or masking their presence. Prey attraction: certain species, such as stomiids, use photophores as lures to attract prey toward their mouths. Intraspecific communication and recognition and counterillumination: some fish use bioluminescence to blend in with light from the surface or within the deep scattering layer. The morphological and ultrastructural analysis of photophores thus provides detailed information about their structure and function. Such studies are crucial for understanding the ecological role of these organisms, particularly in relation to marine food web dynamics. Bioluminescent organisms, such as plankton, are in fact fundamental to the food chain and can act as indicators of environmental health, signaling changes such as eutrophication or pollution.

## 1. Introduction

Dragonfishes belonging to the family Stomiidae (approximately 28 genera and 287 species) live in the mesopelagic and bathypelagic waters of the warm and temperate zones of all oceans [1]. They have an elongated and darkly pigmented body and are characterized by various specializations and adaptations to life at depths among which the presence of a sophisticated system of light organs (photophores) stands out. Overall, four species of stomiid fishes have been reported in the Mediterranean Sea: *Bathophilus nigerrimus* (Giglioli, 1882), *Borostomias antarcticus* (Lönnberg, 1905), *Stomias boa boa* (Risso, 1810), and *Chauliodus sloani* (Bloch and Schneider, 1801) [1,2,3,4]. Among Mediterranean species, *C. sloani* is an important component of the pelagic fish community [1] and normally lives at 400 m depth in the deep scattering layer (DSL). In the Strait of Messina (Central Mediterranean Sea), especially in the upwelling area, *C. sloani* can also be found in surface waters and, periodically, stranded specimens have been observed on the coast [5,6,7]. Furthermore, *C. sloani*, in the Strait of Messina, is present as prey in the diet of several marine predators, such as tunas, especially during the pre-spawning and gamete deposition period in the area of the Strait of Messina and in the Eastern Mediterranean Sea [8,9]. Dragonfishes are abundant in the ocean and contribute significantly to the transfer of energy within marine ecosystems through their diel vertical migration [10,11]. They rise to the upper ocean layers at night to hunt prey such as zooplankton and small micronekton, then descend back to deeper waters during the day [8,9,12,13]. In the Mediterranean, in order to broaden the current knowledge on this species [3,14,15], we have studied the structure of the ventral light organs, offering some considerations on its camouflage and predation strategy. Photophores are specialized glandular organs located on the body surface of various marine animal species. These organs are specialized in the production of bioluminescent light, which is emitted as a result of specific chemical reactions [16,17,18,19,20]. Photophores are formed by a purely glandular portion represented by the tank, where the photocytes are grouped and by the lens filter, inside which the light reaction occurs. Various dioptric annexes complete the structure and support the bioluminescent function. Specifically, the reflector and the pigmented layer, rich in melanin granules, which envelop the entire organ, orientate the direction of light rays and prevent their dispersion, and the gelatinous layer, which is located in the distal portion of the photophore, also assumes a protective function for the entire organ [21,22,23,24,25,26,27,28]. Photophores are common among fish inhabiting mesopelagic and deep-sea zones, and are found in several families [1]. Typically, photophores are arranged along the ventral or ventral–lateral surfaces of the animal’s body, and in regions such as the head or caudal peduncle [12,29]. Their main function, particularly when located on the ventral or ventral–lateral surfaces, is to generate counterillumination, which reduces the visibility of the fish’s silhouette to potential predators, thereby enhancing camouflage [14]. The findings from this study represent a useful starting point for further investigation into the physiology, function, and ecological role of the photogenic system of *C. sloani* in its natural mesopelagic environment.

## 2. Materials and Methods

A total of 5 specimens of *Chauliodus sloani* were found stranded in the Sicilian coast of the Strait of Messina (38°15′ N, 15°39′ E), Central Mediterranean Sea, due to the upwelling currents that characterize this study area [5]. When fish samples were collected on the shore, they were still alive or recently dead and photophores were still active. Samples were immediately fixed.

### 2.1. Sample Treatment

Three fresh specimens were fixed in 4% paraformaldehyde and treated with routinary procedures for paraffin embedding in dorsal–ventral view [30,31,32,33,34]. From included tissues, 7 μm thick serial sections were obtained. Deparaffinized and rehydrated slices were stained with hematoxylin and eosin (H&E) (Carazzi’s Hematoxylin Nuclear staining, (05-M06012 Bio-Optica s.p.a., Milan, Italy); Eosin Y 1% aqueous solution cytoplasmic staining, (05-M10002, Bio-Optica s.p.a., Milan, Italy) and Masson’s Trichrome with aniline blue method (04-010802, Bio-Optica s.p.a. Milan, Italy). Finally, stained sections were examined under a Leica DMRB light microscope equipped with a Leica MC 120 HD camera (Leica Application Suite LAS V4.7, Leica Microsystems GmbH, Wetzlar, Germany).

### 2.2. Histochemistry

To ascertain the biochemical nature of the photophore structure, a histochemical analysis was conducted on a deparaffinized and rehydrated serial section. The secretory or stromal nature of mucins was detected using Alcian Blue Periodic acid Schiff’s (Ab-PAS) (04-163802, Bio-Optica) and, Mercury-Bromophenol Blue Method (Hg-BPM). These methods have been applied to identify glycoproteins (magenta red stained) and protein granules (blue stained), respectively.

### 2.3. Ultrastructure Analysis

For the ultrastructural study, 2 fresh specimens were fixed in 2.5% glutaraldehyde in 0.1 M phosphate buffer (pH 7.4) at +4 °C, washed with 0.1 M phosphate buffer (pH 7.4), postfixed in 1% OsO_4_ in 0.2 M phosphate buffer (pH 7.4) at +4 °C for 1 h, dehydrated in graded ethanol, and immersed in propylene oxide. Then, the tissues were dehydrated with increasing alcohol concentrations. The dehydrated pieces were embedded in Durcupan (Sigma–Aldrich/Fluka, St. Louis, MO, USA). Ultrathin silver–golden sections were cut with a diamond knife on a Reichert Jung Ultracut E, placed on uncoated 200-mesh copper grids, contrasted with methanolic uranyl acetate and lead citrate, and photographed with a JEOL JEM-100 SX transmission electron microscope at 80 kV (JEOL USA, Inc., Peabody, MA, USA) [35].

### 2.4. Immunohistochemistry

To demonstrate the localization of nNOS in *C. sloani*, serial sections were deparaffined and rehydrated, rinsed several times in Phosphate-Buffered Saline (PBS) 0.1 M pH = 7.4, and incubated in 0.3 % H_2_O_2_ (PBS) solution for 3 min to prevent the activity of endogenous peroxidase, and finally blocked in 25% bovine serum albumin (BSA) for 1 h. Representative sections were incubated overnight at 4 °C in a humid chamber with polyclonal rabbit anti-nNOS/NOS Type I (1:250) (BD Transductions Laboratories^TM^ cat. n. 610310, BD Biosciences, San Jose, CA, USA). After incubation, sections were washed in the same buffer and incubated for 90 min at room temperature with Alexa Fluor^®^ 594 goat anti-rabbit IgG antibody (1:100) (Molecular Probes, Invitrogen A11012, Invitrogen, Waltham, MA, USA). After washing, sections were mounted with Fluoromount Aqueous Mounting Medium (Sigma Aldrich, St. Louis, MO, USA) to prevent photobleaching and were cover slipped. In order to reduce photodegradation, each image was quickly captured. Sections were analyzed and images acquired using a ZeissLSMDUO confocal laser scanning microscope with META module (microscope LSM700 AxioObserver, Carl Zeiss Micro Imaging GmbH, Jena, Germany) equipped with a 555 nm laser line, and fluorescence emission was collected using the spectral detector set to a bandwidth of 600–650 nm, based on the fluorophore’s emission spectrum. The pixel dwell time was set to 2.0 µs/pixel, with line averaging of 2× to improve signal quality. The pinhole was set to 1 Airy unit. Detector gain and laser power were optimized to minimize photobleaching and maximize signal-to-noise ratio and were kept constant across all samples within each experimental condition.

## 3. Results

**Figure 1:**
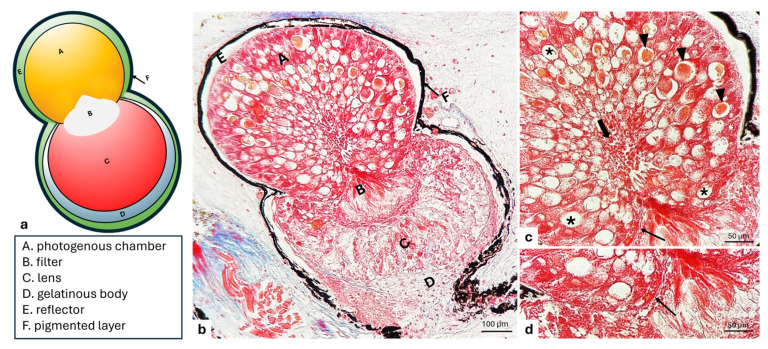
*C. sloani* photophore. (**a**) A graphical representation. (**b**) A light micrograph of a longitudinal section through the skin of an entire photophore: (A) photogenous chamber, (B) filter, (C) lens, (D) gelatinous body, (E) reflector, and (F) pigmented layer. (**c**) Cells with a radial organization, vesicles with granules (arrow heads), vacuoles (asterisks), basal lamina (thin arrow), and extruded granules into the lumen (thick arrows). (**d**) Detail of the above photographed photogenic tissue (A) and filter (B) separated by an evident basal lamina (arrow). Masson’s trichrome with aniline blue stain. Magnification (**b**) 20×, (**c**,**d**) 40×.

The histological results allow us to observe bilobate-shaped photophores of *Chauliodus sloani* situated within the skin. Each photophore shows the presence of functionally different parts, a glandular photogenous chamber called a tank, a filter, a lens, and a series of dioptric annexes, such as a basal gelatinous body, and an adjacent ensheathing reflector enclosed in a spherical layer of pigmented cells, separated from the epidermis by a layer of connective tissue. The photogenous chamber exhibits an ampullar shape occupying the proximal part of the photophore, at the opening of which a filter is situated. The last distal part consists of a bigger lobe, where we find a filter and a lens (Figure 1a,b). In detail, the photogenic tissue contained in the photogenous chamber appears to be constituted by polyhedral photogenic cells, different in size and elongated in shape, with a large base showing a prominent nucleus and a tapered apical portion rich in several granules. The cytoplasm is granular, extremely vesiculated, vacuolated, and intensely basophilic. With a radial arrangement, the cells appear to be grouped in series and converging towards the central part, where they release secretory granules into a lumen (Figure 1b,c). The deeper photogenic cells placed on a basal lamina appear smaller than other cells of the photogenous chamber (Figure 1c,d). The apex of the photogenic cells are directed toward the filter of the photophore (Figure 1a–c).

**Figure 2:**
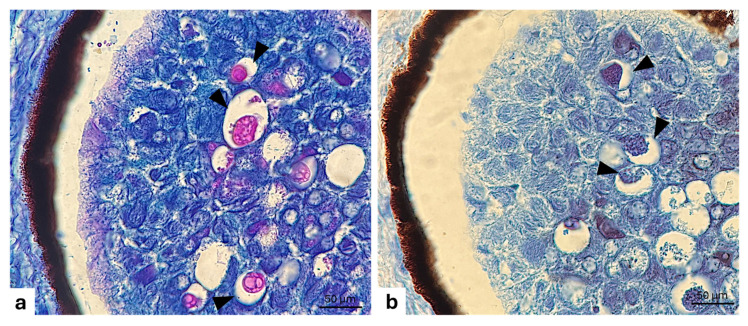
Longitudinal section of photogenous chamber of *C. sloani* photophore. (**a**) Vesicles with glycoprotein granules (arrow heads); (**b**) vesicles with protein granules (arrow heads). (**a**) Alcian blue PAS stain; (**b**) Hg BPB (Mercury Bromine Phenol Blue) stain. Magnification 40×.

The use of a series of histochemical techniques and of histological staining procedures highlights the glycoprotein nature of the photocyte granules (Figure 2a,b).

**Figure 3:**
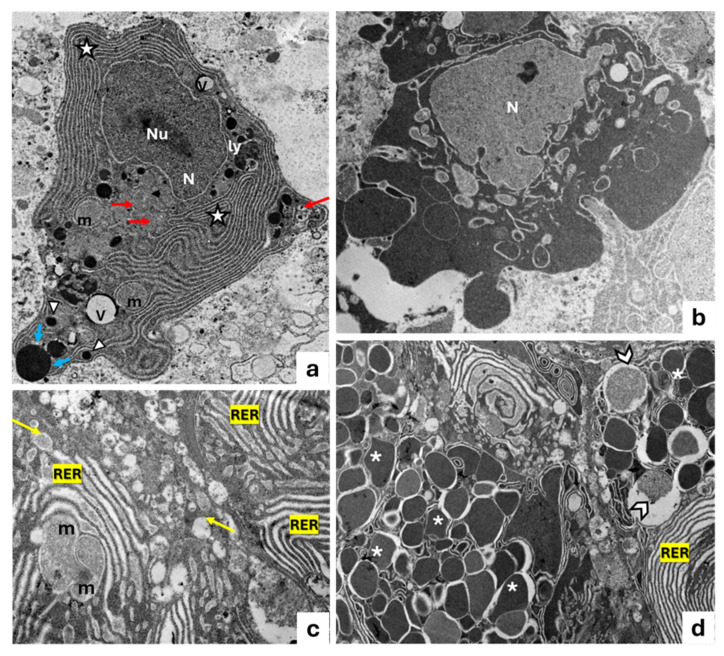
Transmission electron micrograph of photocytes of *C. sloani.* (**a**) Note a wide rough endoplasmic reticulum (RER) (stars) around the basal nucleus (N), evident nucleolus (Nu), secretory vesicles (red arrows), vacuoles (V), and lysosomes (ly); in the apical portion, there are some secretion granules (arrowheads) with one in extrusion phase (blue arrows). (**b**) Note a degenerating photocyte with pyknotic nucleus (N). (**c**) Occurrence of mitochondria (m) with evident mitochondrial crest vesicles (yellow arrows) and RER. (**d**) Vesicles during the filling phase (gallon arrows), presence of granules of different electronic density with light-colored halos (asterisk), and RER. Magnification: (**a**) 2000×, (**b**) 5000×, (**c**) 2500×, (**d**) 5000×.

According to electron transmission microscopy (TEM), the smaller photogenic cells display a wide basal cytoplasm containing an ovoidal nucleus, with an evident nucleolus, as well as mitochondria, lysosomes, secretory vesicles, vacuoles, and a rough endoplasmic reticulum (RER) present throughout the cytoplasmic area. The RER consists of cisterns, regular in size, situated parallel to one another and concentric to the nucleus, while their narrow apex is full of secretion granules, some of which are observed during extrusion (Figure 3a). Some secretory cells undergoing a degeneration process with pyknotic nuclei extruding their secretion are observed (Figure 3b). The larger photogenic cells exhibit numerous mitochondria with evident mitochondrial crests, many vesicles, vacuoles, abundant RER, scattered ribosomes, and several granules (rimmed by *light-colored halos*) of different sizes and electronic densities. Moreover, vesicles during the filling phase with a gradual accumulation of glycoprotein material are also evident (Figure 3c,d).

**Figure 4:**
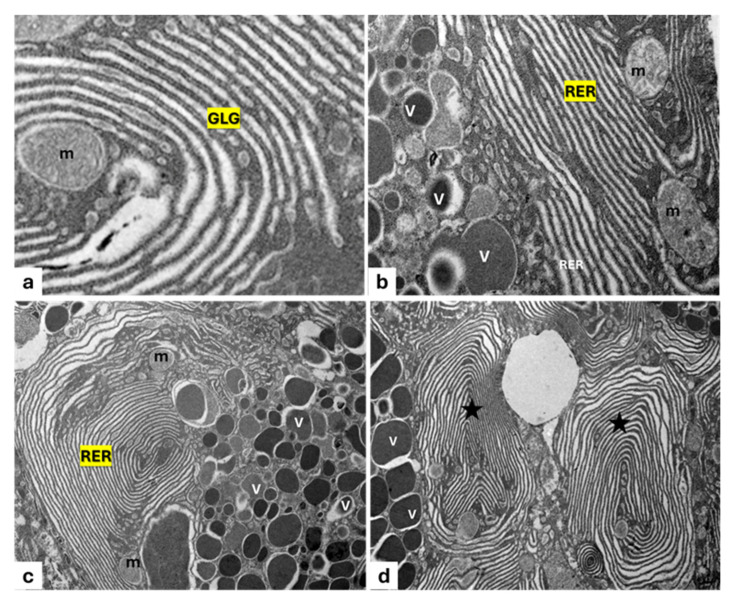
Transmission electron micrograph of a photogenic cell of *C. sloani*. (**a**) Evident Golgi apparatus (GLG), mitochondrion (m); (**b**) rough endoplasmic reticulum (RER) with regular-in-shape cisterns, secretory vesicles (V), mitochondria (m); (**c**) organization of the RER into concentric lamellar membranes with mitochondria (m), secretory vesicles (V); (**d**) occurrence of concentric membranous whorls (stars), secretory vesicles (V). Magnification: (**a**) 1000×, (**b**) 6000×, (**c**) 10,000×, (**d**) 4000×.

Most of the vesicles that are observed are derived from the Golgi apparatus (Figure 4a). In addition, the RER exhibits cisterns, regular in shape, arranged in parallel with each other (Figure 4b). Moreover, the cisternal membranes, undergoing wide sequential infoldings to develop concentric lamellar structures, are seen. Some of these appear to be provided with material of varying density and containing cytoplasmic cores with mitochondria (Figure 4c). Frequently, some concentric lamellar membranous structures with a spiral layout gradually change into whorl forms. The proximity of some secretory vesicles of different sizes with lamellar components is also evident (Figure 4b–d).

**Figure 5:**
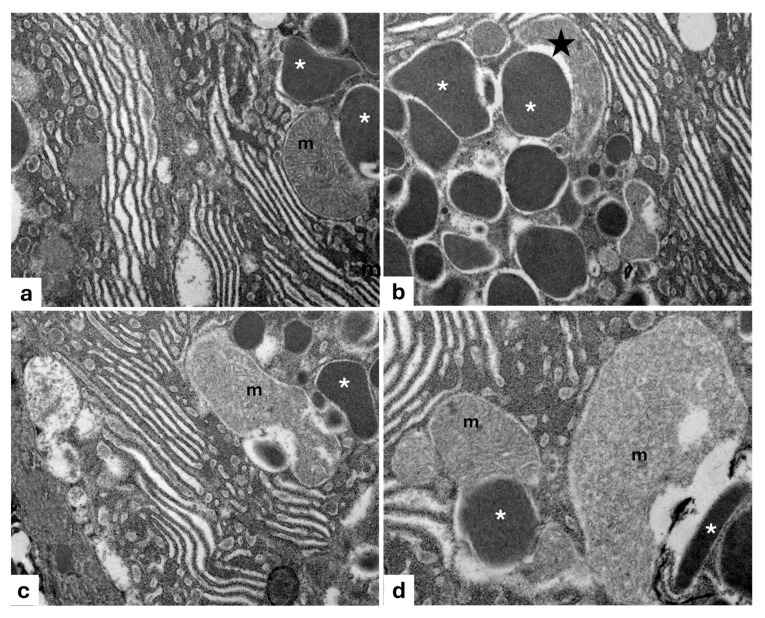
Transmission electron micrograph of photocytes of *C. sloani*. Heterogeneity among mitochondria (m) near granules (asterisks) can be observed. (**a**) A mitochondrion with evident mitochondrial crests; (**b**) an arch-shaped mitochondrion (star); (**c**,**d**) mitochondria (m) filled with an amorphous product (asterisk). Magnification 6000×.

Abundant long mitochondria with evident mitochondrial crests close to the granules (Figure 5a) are present, some of which appear further stretched, exhibiting structural changes such as a depressed outline and/or outline with arch-shaped invaginations (Figure 5b); these depressions, more or less deep, are filled with a seemingly amorphous content similar to the mature secretion product (Figure 5c,d).

### 3.1. Filter

**Figure 6:**
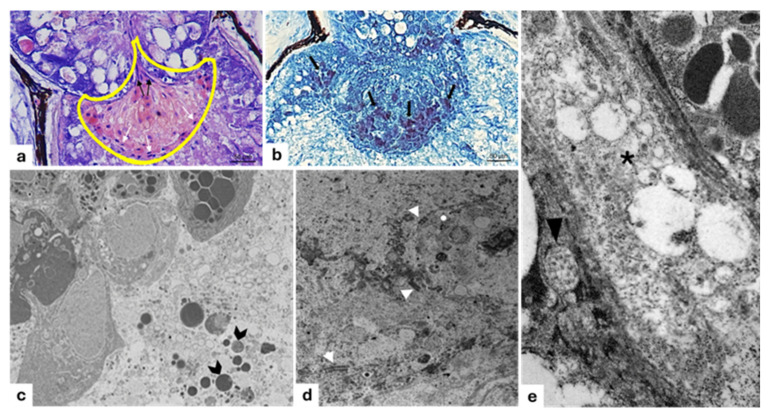
Light and transmission electron micrograph of a longitudinal section of filter of *C. sloani* photophore. (**a**) The yellow-outlined area indicates the filter with typical pear-shaped, elongated cells (black arrows), flattened cells (white arrows); (**b**) occurrence of granules of proteinaceous nature (arrows); (**c**) photocyte-extruded secretion granules (gallon arrows); (**d**) note desmosomes along the cell borders (arrow heads); (**e**) interstitial unmyelinated nerve ending (asterisk) containing many synaptic vesicles having dense cores (arrowhead). (**a**) Hematoxylin/eosin stain; (**b**) Hg BPB (Mercury Bromine Phenol Blue) stain. Magnification (**a**) 40×; (**b**) 20×; (**c**) 2500×; (**d**) 10,000×; (**e**) 6000×.

**Figure 7:**
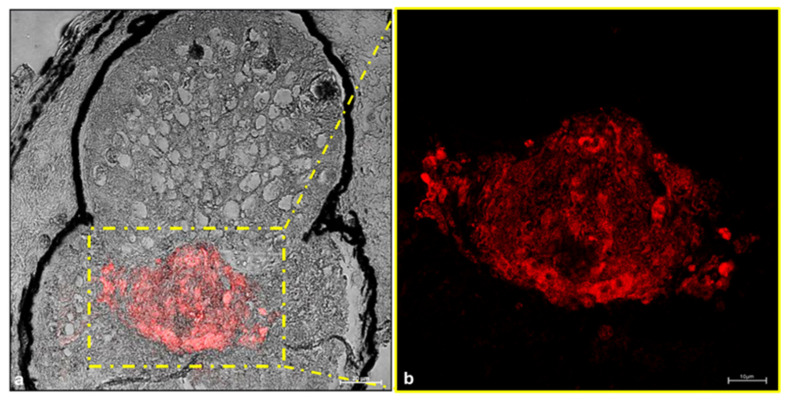
Immunohistochemical detection (immunofluorescence method) of nitric oxide synthase (nNOS) in *C. sloani* photophore. (**a**) Transmitted light of nNOS immunoreactivity. The yellow-outlined area indicates the nNOS immunopositive filter; (**b**) high magnification of nNOS immunoreactivity in the filter; the cytoplasm of the filter cells showed NOS immunopositivity. Magnification: (**a**) 20×, (**b**) 40×.

In the light microscopical investigation, the filter, overlying the photogenous chamber, exhibited a typical pear-shaped projection toward the lens. The innermost region of the filter contains central polyhedral cells arranged in rows. External cells laying in the distal portion of the filter body are flattened and bordered by a basal lamina, thus forming a pavement-like layer over the body of the lens (Figure 6a and 7b). The cells are characterized by rounded eccentric nuclei and a dense cytoplasm with granules (Figure 6a) identified, also, by histochemical stains (Figure 6b). Filter cells which lie closely adjacent to photogenic cells are elongated (Figure 6a) and organized in order to collect the photocyte secretum, as observed by TEM (Figure 6c). Different histochemical stains show the glycoprotein nature of granules. The TEM investigation also reveals that the cells of the filter appear closely united by well-defined desmosome adhesions (Figure 6d). Interstitial unmyelinated nerve endings between two cells show synaptic vesicles with dense cores (Figure 6e).

#### Immunohistochemistry

The results of the immunohistochemical investigation conducted to ascertain the presence of nNOS as a modulator in adrenergic control for the emission of light show that the filter cells of *C. sloani* are nNOS immunopositive (Figure 7).

### 3.2. Lens

**Figure 8:**
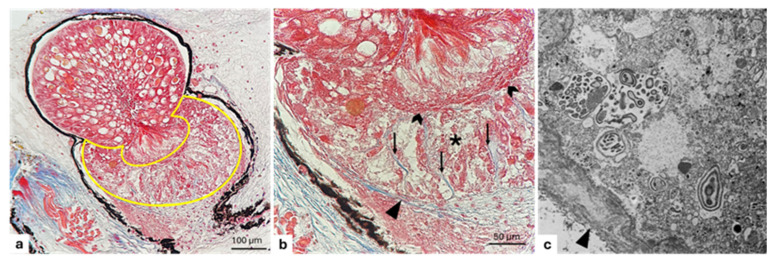
Light and transmission electron micrographs of lens of *C. sloani* photophore. (**a**) The yellow-outlined area indicates the lens; (**b**) elongated lens cells with granular cytoplasm and mucoid appearance (asterisk), lens cells ordered in parallel series supported by abundant connective tissue (arrows), evident basal membrane (arrowheads), filter cell basal lamina (gallon arrows); (**c**) basal membrane (arrowhead). (**a**,**b**) Hematoxylin/eosin stain. Magnification: (**a**) 20×; (**b**) 40×; (**c**) 1500×.

In the optical microscopy investigation, the lens, lying over the filter, exhibits elongated cells with a granular cytoplasm that is not very dense. The cells appear organized in parallel series supported by connective tissue abundant throughout the area (Figure 8a,b). A prominent basal lamina separates lens cells from the surrounding connective tissue, as also seen with TEM (Figure 8c).

**Figure 9:**
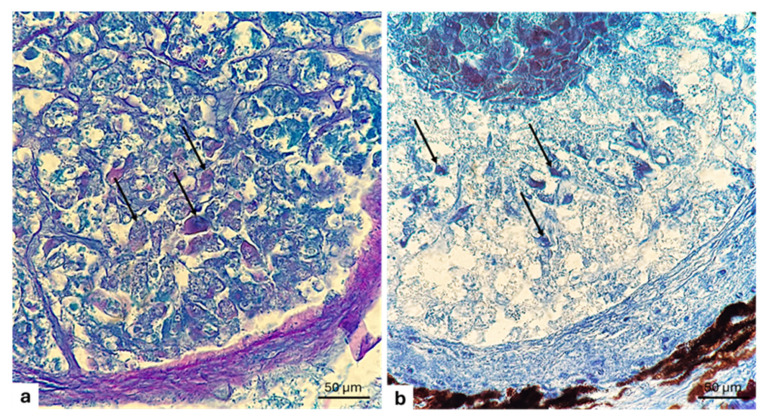
Longitudinal section of lens of *C. sloani* photophore. (**a**) Secretory granules of glycoprotein nature (thin arrows); (**b**) secretory granules of protein nature (thin arrows); (**a**) Alcian blue PAS stain; (**b**) Hg BPB (Mercury Bromine Phenol Blue) stain. Magnification: 40×.

The lens cell, like in the photogenous chamber, contains secretion granules of glycoprotein, as confirmed by histochemical stains (Figure 9a,b).

**Figure 10:**
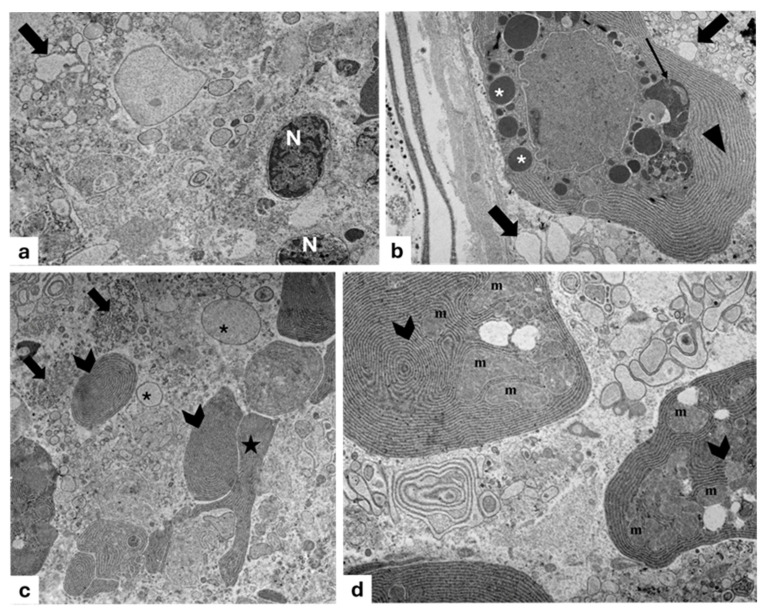
Transmission electron micrograph lens of *C. sloani* photophore. (**a**) Cytoplasm of lens cell rich in smooth endoplasmic reticulum with a tubular arrangement (arrow), presence of nuclei (N); (**b**) continuity between the membranes of the granular (arrow head) and smooth endoplasmic reticulum (arrows), lysosomes (thin arrow), protein granules (asterisks); (**c**) presence of cisternal membranes having shape of skeins (gallon arrows) or loops (star), smooth endoplasmic reticulum (arrows), and lipid granules (asterisks); (**d**) mitochondria (m) included in the lamellar system (gallon arrows). Magnification: (**a**–**c**) 2000×, (**d**) 5000×.

Under TEM observation, the cells exhibit an evident nucleus, and their cytoplasm displays a compacted dense granular and mucoid appearance with a conspicuous smooth endoplasmic reticulum (REL) associated with an abundant RER. The cisternal membranes of the REL display a tubular arrangement (Figure 10a–c). In addition, some of the cisternal membranes of the RER are characterized by particular lamellar formations, pseudo-myelinic bodies, similar to loops or skeins. Numerous multivesicular bodies and mitochondria included in the lamellar system are also seen. Lysosomes and mitochondria, scattered in the cytoplasm, and several lipid and protein granules are also observed (Figure 10a,b).

### 3.3. Gelatinous Body

**Figure 11:**
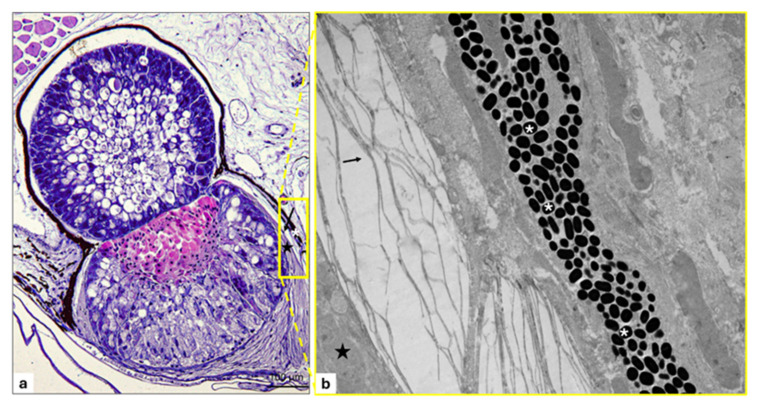
Light and electron micrograph of a longitudinal section of *C. sloani* photophore. (**a**) The yellow-outlined area indicates the dioptrical annexes: gelatinous body (star), reflector (thin arrow), pigmented layer (asterisk); (**b**) mucous appearance of gelatinous body (star). Thin arrow points to empty spaces left by guanine crystal, asterisks indicate the pigmented layer rich in melanin granules. Magnification: (**a**) 20×, (**b**).

The gelatinous body located in the anterior part of the photophore, distally to the lens and laterally surrounded by the reflector walls, is observed. The light microscopical investigation shows a mucous appearance and consists of large and elongated cells, tightly adjoined to each other, having rounded and evident nuclei (Figure 11a). The ultrastructural analysis of these cells shows a cytoplasm containing mucoid material (Figure 11b).

### 3.4. Reflector

The reflector covers the entire photophore. Through the light microscopical investigation, it appears as a dense layer of connective tissue with stacked and elongated cells regularly scattered and orientated tangentially to the curve of the photophore (Figure 11). The TEM investigation shows a lamellar structure provided with elongated connective tissue cells embedded in an amorphous matrix. These cells appear similar to a mesh net. Within the cells, the empty parallel spaces once occupied by the guanine crystals are now visible because of the contrast medium used (Figure 11b).

### 3.5. Pigmented Layer

In the external surface of the reflector, the pigmented layer, under light microscopical investigation, is observed. The latter appears as a dark layer characterized by copious melanin granules, particularly visible in the ultrastructural view (Figure 11a,b).

## 4. Discussion

This study explores the structural and ultrastructural aspects of the ventral photophores of *Chauliodus sloani*, suggesting that bioluminescence in this species is of glandular origin, as has already been observed in other fish species [6,22,29,36,37,38,39]. A microscopic investigation revealed that the photocytes within the photogenous chamber produce secretion granules of a protein and glycoprotein nature, which are expelled at maturity through active exocytosis. The secretion is subsequently transported into the filter via a dedicated channel. The ultrastructural analysis indicated the protein and glycoprotein nature of the granules, further supported by the abundant presence of rough endoplasmic reticulum (RER) within the cells, indicating intense protein synthesis, along with the presence of smooth endoplasmic reticulum (SER). These elements suggest that the photocytes produce secretions that act on surrounding cells for the subsequent transduction of the light signal. Moreover, the photocytes involved in significant secretory activity display two different aspects of the rough endoplasmic reticulum: the first is dedicated to the production of glycoprotein substances, as evidenced by the presence of numerous ribosomes; the second forms concentric structures that Bassot [40] referred to as parasomes, and the stages of their formation can be gradually followed within the photocytes. The pear-shaped structure known as the filter, as also seen in Myctophidae [41], plays a collection function, receiving the secretory product via a channel connected to the photogenous chamber. The lens, surrounding the filter, appears to be formed by glandular cells, particularly rich in endoplasmic reticulum, indicating their important role in the glycoprotein secretory activity. Moreover, numerous nerve endings were identified in this structure. This evidence, along with the nNOS immunoreactivity in the filter cells, suggests that the bioluminescent reaction is primarily processed within the lens–filter complex. Specifically, synthesized nitric oxide (NO), derived from L-arginine, acts as a neurotransmitter, modulating the adrenergic control of bioluminescence in response to external stimuli. In the photophores of *C. sloani*, both a reflector and a pigmented layer are also present. These two components serve both dioptric and protective functions for the entire photophore [21,22,23,42]. The reflector varies greatly depending on the species; for instance, in Myctophidae, it can consist of bundles of filaments and guanine platelets, as in *C. maderensis* [39,43], whereas *D. holti* exhibits a single layer of filaments [29]. Other species, such as *A. hemigymnus* and *M. muelleri* (Sternoptychidae), have a reflector structure similar to that observed in *C. sloani*, with guanine-like platelets arranged in precise patterns [44,45]. This is also supported by Strum [46], who noted that guanine may dissolve, leaving empty spaces within the reflector. The pigmented layer, on the other hand, appears as a region where melanin granules accumulate, enveloping the entire organ except for its distal portion, which is covered by the gelatinous body. This structure is present in the photophores of many and serves both a dioptric function, by preventing the dispersion of the light reaction already concentrated by the reflector, and a protective function for the organ as a whole [29,36,37,38,39]. The gelatinous body marks the distal boundary of the ventral photophores in *C. sloani*. This structure is not always observed in the luminous organs of other species; in fact, it is absent in some species of Myctophidae [39,47], while present in others [29]. It can also be found in species belonging to Sternoptychidae [37] and Stomidae [48]. The function of this accessory is undoubtedly dioptric [40,49], as it is composed of cellular components rich in mucoid substances that make them refractive.

## 5. Conclusions

The structural and ultrastructural analysis of the ventral photophores in *Chauliodus sloani* suggests a glandular origin of bioluminescence, as also shown by Mallefet and colleagues, showing the presence of Adrenalin, Noradrenalin, Isoprenaline, and Phenylephrine [50]. Photocytes within the photogenous chamber exhibit intense secretory activity, producing proteinaceous and glycoprotein granules that are exocytosed and conveyed through a specialized channel to the lens–filter complex. The presence of both rough and smooth endoplasmic reticulum, along with Golgi apparatus and parasome structures, underscores the photocytes’ active role in synthesis and processing of luminescent substances. The lens–filter complex emerges as a key site for light modulation and neurochemical control, supported by immunohistochemical evidence of nNOS activity and the known regulatory function of nitric oxide in bioluminescent pathways. Although interdisciplinary studies are needed to clarify how the light produced is emitted, based on known data in other species, one might think that the reflector and pigmented layer contribute both to the directionality and optical insulation of the emitted light, while the presence of a gelatinous body at the distal end composed of refractive, mucoid-rich elements suggests a dioptric function. Collectively, these findings provide new insights into the specialized architecture of bioluminescent organs in *C. sloani.* Moreover, future studies are needed to identify the glycoprotein products secreted by the cells of the photogenous chamber, filter, and lens to clarify the biochemical role of each of these structures in bioluminescence.

## Data Availability

All data presented in this study are available from the corresponding author upon responsible request.
